# Stability of total phenolic concentration and antioxidant capacity of extracts from pomegranate co-products subjected to in vitro digestion

**DOI:** 10.1186/s12906-016-1343-2

**Published:** 2016-09-13

**Authors:** Olaniyi Amos Fawole, Umezuruike Linus Opara

**Affiliations:** 1 South African Research Chair in Postharvest Technology, Department of Horticultural Science, Stellenbosch University, Private Bag X1, Stellenbosch, 7602 South Africa; 2 South African Research Chair in Postharvest Technology, Department of Food Science, Stellenbosch University, Private Bag X1, Stellenbosch, 7602 South Africa

**Keywords:** By-product, DPPH, Polyphenols, Pomegranate, Pepsin, Value-addition

## Abstract

**Background:**

Co-products obtained from pomegranate juice processing contain high levels of polyphenols with potential high added values. From value-addition viewpoint, the aim of this study was to evaluate the stability of polyphenolic concentrations in pomegranate fruit co-products in different solvent extracts and assess the effect on the total antioxidant capacity using the FRAP, DPPH*˙* and ABTS^+^ assays during simulated in vitro digestion.

**Methods:**

Pomegranate juice, marc and peel were extracted in water, 50 % ethanol (50%EtOH) and absolute ethanol (100%EtOH) and analysed for total phenolic concentration (TPC), total flavonoids concentration (TFC) and total antioxidant capacity in DPPH˙, ABTS^+^ and FRAP assays before and after in vitro digestion.

**Results:**

Total phenolic concentration (TPC) and total flavonoid concentration (TFC) were in the order of peel > marc > juice throughout the in vitro digestion irrespective of the extraction solvents used. However, 50 % ethanol extracted 1.1 to 12-fold more polyphenols than water and ethanol solvents depending on co-products. TPC and TFC increased significantly in gastric digests. In contrast, after the duodenal phase of in vitro digestion, polyphenolic concentrations decreased significantly (*p* < 0.05) compared to those obtained in gastric digests. Undigested samples and gastric digests showed strong and positive relationships between polyphenols and the antioxidant activities measured in DPPH, ABTS^+^ and FRAP assays, with correlation coefficients (*r*^2^) ranging between 0.930–0.990. In addition, the relationships between polyphenols (TPC and TFC) and radical cation scavenging activity in ABTS^+^ were moderately positive in duodenal digests.

**Conclusion:**

Findings from this study showed that concentration of pomegranate polyphenols and the antioxidant capacity during in vitro gastro-intestinal digestion may not reflect the pre-digested phenolic concentration. Thus, this study highlights the need to provide biologically relevant information on antioxidants by providing data reflecting their stability and activity after in vitro digestion.

## Background

Several epidemiological and intervention studies have reported a direct relationship between consumption of fresh fruits and vegetables, and prevention of most degenerative diseases as well as slowing of the ageing process [1]. Fruits and vegetables are rich in polyphenols, which do not only play physiological roles in plants but also act as antioxidants by donating a hydrogen atom or an electron to other compounds, scavenging free radicals, quenching singlet oxygen, and maintaining a balance between oxidants and antioxidants to improve human health [2, 3]. The antioxidative phytochemicals, especially phenolic compounds, found in pomegranate (*Punica granatum* L.) fruit have received increasing attention for their potential role in the prevention of human diseases [4–6]. The phenolic content of pomegranate is usually influenced not only by the cultivar but also depends on the fruit fraction [4, 7]. Phenolic compounds such as ellagitannins, punicalagin and punicalin are found in the juice, however, most of the phenolic compounds are mainly located in the fruit peel and mesocarp [4, 7]. Although very few industrial processing techniques allow the introduction of pomegranate peel extract into the juice, most juice extraction techniques involve direct disposal of co-products of commercial juicing as waste or for limited purposes such as cattle or pig feed [8, 9]. The co-products include fruit peel and the residual material (seed and aril membrane) called marc. Often times, disposal of these co-products represent a problem for management, contamination, and environmental issues. Interestingly, phenolic compounds such as punicalagins contained in pomegranate peel, when released into the juice, gives the outstanding antioxidant activity and strongly influence the nutritive value of the juice and are wholly or partially responsible for possible therapeutic effects observed in some commercial pomegranate juice [4]. The results from our previous studies also showed that the peel had the higher content of antioxidants than the juice and could be a good source for producing high-value antioxidants and other chemotherapeutic agents [6]. Moreover, the choice of solvent in extracting polyphenols from pomegranate fruit co-products has also been reported to influence the quantity and total antioxidant capacity of the extracted polyphenols [10–12]. From the agro-industrial and health perspectives, the co-products obtained from pomegranate juice processing contain high levels of polyphenols with high added values. Therefore, the recovery of valuable compounds from these co-products are beneficial. There has been extensive research into the antioxidant capacity of different co-products of pomegranate fruit and co-products [8, 9]. While it may be useful to know the antioxidant capacity of pomegranate co-products relative to the juice before digestion for comparative purposes, this is not a true reflection of the potential health benefits. A more realistic view is the antioxidant capacity of a given sample which has been subjected to simulated in vitro digestion procedure, when the antioxidants potentially available for absorption can be measured [13].

The potential availability of antioxidants after digestion is an important initial measure. For instance, previous studies have shown that the bioavailability of certain phenolic compounds in pomegranate and orange juices is poor, resulting in limited effect on health [14, 15]. Hence, the most important factors in determining the potential beneficial effects of polyphenols on the gut epithelial cells are their stability under gastro-intestinal conditions. From value-addition viewpoint, the aim of this study was to evaluate the stability of total phenolic content of pomegranate fruit juice and co-products from (marc and peel) in different solvent extracts and assess the effect on the total antioxidant capacity using the FRAP, DPPH˙ and ABTS^*+*^ assays during simulated in vitro digestion.

## Methods

### Plant materials

**Fig. 1:**
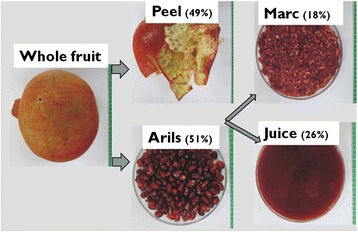
Proportion (%) of different fruit co-products of pomegranate (cv. Kessari). Data showing percentage proportions of each co-product per whole fruit weight

Pomegranate fruit (cv. Kessari) were harvested from an orchard in Ladismith (33°29′S 21°16′E) Western Cape, South Africa. Fruit were verified by Mr. Mashavhathakha of the Agricultural Research Council, Stellenbosch. Harvested fruit at commercial maturity were transported to the postharvest laboratory at Stellenbosch University and a voucher specimen was retained with as POM.K2013. In triplicates, ten healthy fruit were washed to remove sand or dirts. Peel fraction was obtained after manual peeling, juice from extracted arils using a blender (Mellerware, South Africa), and the resultant residue from juicing was collected as the marc. Figure 1 illustrates the pomegranate fruit portions analysed in this study. Fruit juice was kept in clean jars and stored at−20 °C while peel (moisture content = 81 %) and marc (moisture content = 79 %) were dipped into liquid nitrogen, frozen at−80 °C and freeze-dried. Dried samples were ground into powder and stored in airtight containers at 7 °C in the dark until used.

### Preparation of extracts

In triplicates, fruit juice (2 mL) or ground material (1 g) was extracted independently with 10 mL of distilled water, 50 % ethanol, and 100 % ethanol. The mixture was vortexed and sonicated in cold water for 1 h before being centrifuged at 4000 rpm for 10 min at 4 °C. The supernatant was gently collected into a clean tube and stored at−20 °C until analysis.

### Simulated in vitro gastro-intestinal digestion

**Fig. 2:**
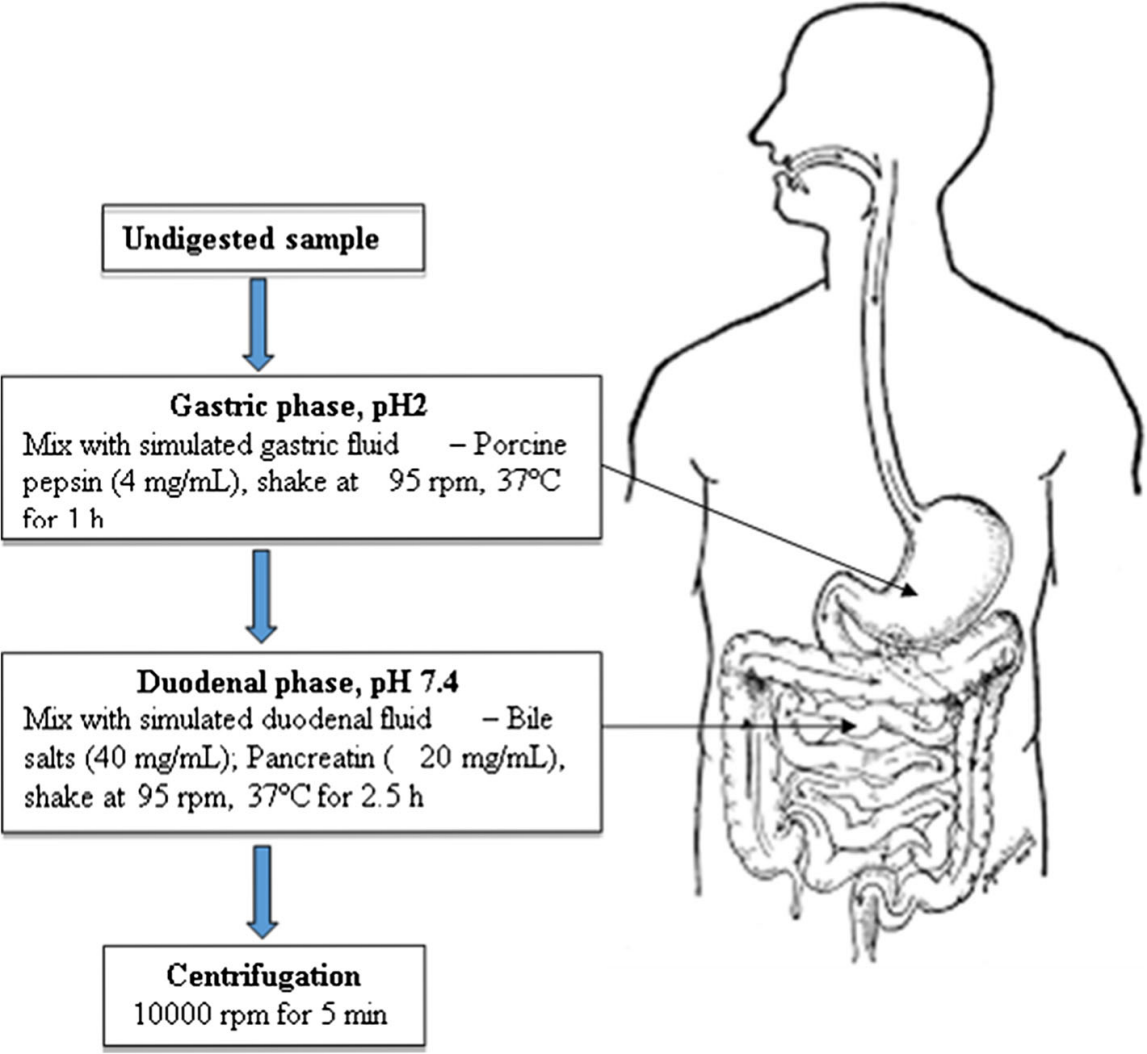
Graphic representation of in vitro gastro-intestinal digestion model carried out with different pomegranate co-products (juice, marc and peel)

In vitro gastro-intestinal digestion model was adapted according to the procedure described by Ryan and Prescott [13] as indicated in Fig. 2. Each supernatant was mixed in 1:5 dilutions with saline to create a final volume of 20 mL in clean amber bottles. The sample was acidified to pH 2.0 with 1 mL of porcine pepsin preparation (0.04 g pepsin in 1 mL 0.1 M HCl) and incubated at 37 °C in a shaking water bath at 95 rpm for 1 h. After gastric phase of the in vitro digestion process, the pH was increased to 5.3 with 0.9 M sodium bicarbonate solution followed by the addition of 200 μL of bile salts glycodeoxycholate (0.04 g in 1 mL saline), taurodeoxycholate (0.025 g in 1 mL saline), taurocholate (0.04 g in 1 mL saline), and 100 μL of pancreatin (0.04 g in 500 μL saline). The pH of each sample was increased to 7.4 with 1 M NaOH, followed by incubation at 37 °C in a shaking bath at 95 rpm for 2.5 h to complete the duodenal phase of the in vitro digestion process. Blanks were prepared with identical chemicals but without test samples and treated under the same conditions as the samples. Undigested samples as well as gastric and duodenal digests were centrifuged (10000 rpm for 5 min) and stored at−80 °C and analysed within 2 weeks.

### Determination of total polyphenols

#### Total phenolic concentration

Total phenolic content (TPC) was determined for undigested samples, gastric and duodenal digests in triplicate by the Folin-Ciocalteu colorimetric method by Makkar [16], with slight modifications [17]. Briefly, extract (50 μl) was mixed with 450 μl of 50 % methanol followed by the addition of 500 μl Folin–C and then sodium carbonate (2 %) solution after 2 min. The mixture was vortexed and absorbance read at 725 nm using a UV–visible spectrophotometer (Thermo Scientific Technologies, Madison, Wisconsin). Results were expressed as gallic acid equivalents (GAE) per 100 mL extracts.

#### Total flavonoid concentration

Total flavonoid concentration was determined as described by Yang et al. [18] with slight modification [17]. Briefly, samples (250 μl) were mixed with 75 μl of 5 % sodium nitrite solution followed by 150 μl of 10 % of aluminium chloride, 500 μl of sodium hydroxide (1 M) and lastly 775 μl of distilled water. The absorbance of the mixture was measured at 510 nm. Results were expressed as catechin equivalents (CE) per 100 mL extract.

### Determination of antioxidant capacity

#### DPPH˙ radical scavenging activity

The DPPH*˙* (2, 2-diphenyl-1-picrylhydrazyl) assay was carried out in triplicate according to the method used by Karioti et al. [19] with some modifications [20]. Briefly, 15 μL of individual samples before and after the gastric and duodenal phases was diluted to create a final volume of 735 μl in test tubes followed by the addition of methanolic DPPH*˙* solution (750 μL, 0.1 mM). The mixture was vortexed and incubated at room temperature for 1 h in the dark, and the decrease in absorbance of DPPH*˙* was measured at 517 nm using a UV–vis spectrophotometer. Absorbance was compared with the standard curve (ascorbic acid; AA = 0−2.0 mM). The free-radical activity of the sample was expressed as ascorbic acid (mM) equivalent per mL or g sample (mM AAE/mL sample).

#### ABTS^+^ radical cation scavenging activity

The ABTS^+^ radical scavenging activity of samples before and after the gastric and duodenal phases was analysed using the method as described by Thaipong et al. [21] with minor modifications. The ABTS^+^ (2, 2′-azino-bis (3-ethylbenzothiazoline-6-sulphonic acid)) working solution containing mixtures of 7.4 mM ABTS^+^ and 2.6 mM of potassium persulfate was freshly prepared and allowed to stand for 12 h at room temperature in the dark to create a stable, dark blue-green radical solution. The working solution was then diluted with methanol to an absorbance of 1.1 ± 0.02 at 734 nm to form the test reagent. Diluted test samples (75 μL) was mixed with 1425 μL of the prepared test reagent and vortexed for 30 s before incubated for 10 min at room temperature in the dark. Absorbance at 734 nm was immediately measured using a UV–vis spectrophotometer. Scavenging activities of test samples were compared with trolox calibration curve, and results were expressed as trolox (mM) equivalent per mL or g sample (mM TE/mL sample).

### Ferric-ion reducing antioxidant power

The antioxidant power of samples at different digestion phases was carried out in triplicate according to the method of Benzie and Strain [22] with a few modifications [20]. The FRAP working solution containing mixtures of 300 mM acetate buffer (200 mL), 10 mM 2,4,6-tripyridyl-s-triazine (TPTZ) (15 mL) and 20 mM ferric chloride (15 mL) was freshly prepared and incubated in a water bath at 37 °C before being used. Individual samples before and after the gastric and duodenal phases (75 μL) were added to 1425 μL of the FRAP working solution before incubation in the dark for 30 min. The reduction of the Fe^3+^-TPTZ complex to a coloured Fe^2+^-TPTZ complex at low pH by extracts was monitored by measuring the absorbance at 593 nm. Trolox (100–1000 μM) was used for the calibration curve, and the results were expressed as trolox (mM) equivalents per mL or g sample (mM TE/mL sample).

### Statistical analysis

All data are presented as mean values (± S.E.). Analysis of variance (ANOVA) was performed, Duncan’s multiple range test was used for mean separation. In addition, where applicable, two-way analysis of variance was conducted using Statistica software (STATISTICA, Vers. 12.0, StatSoft Inc., USA) to determine the effects of solvent extract (factor A) and digestion phase (factor B) on dependent variables. Graphical presentations were made using GraphPad Prism software version 4.03 (GraphPad Software, Inc. San Diego, USA). Furthermore, a Pearson’s correlation analysis was carried out using the statistical XLSTAT software Version 2014.4.01 (Addinsoft, France) to monitor the relationship between total phenolics and antioxidant properties.

## Results and discussion

### Changes in polyphenols after gastric and duodenal phases of in vitro digestion

Total phenolic concentration (TPC) and total flavonoid concentration (TFC) in extracts of the investigated pomegranate co-products and digests are shown in Tables 1 and 2. Overall, the polyphenol concentrations obtained showed high but varying amounts of total phenolics and flavonoids depending on co-products and extraction solvent. TPC obtained from undigested extracts was significantly (*p* < 0.05) higher in 50 % ethanol extracts than water and ethanol extracts, with between 1.1 and 12-fold regardless of fruit co-products (Table 1). Interestingly, however, this is not the case for the quantity of TFC extracted in the undigested samples, as the suitability of solvent to extract the high amount of flavonoids seemed to be dependent on the co-product in question (Table 2). For instance, while there was no significant (*p* > 0.05) difference in TFC obtained from juice extracts, absolute ethanol extracted significantly (*p* < 0.05) higher TFC (282.20 mg CE/100 mL) than water (199.40 mg CE/100 mL) and 50 % ethanol (157.80 mg CE/100 mL) from marc whereas for pomegranate peel, 50 % ethanol extracted the highest amount (1505 mg CE/100 mL) followed by water and 100 % ethanol extracts with 1197 and 844.80 mg CE/100 mL, respectively, in the undigested samples (Table 2). Polyphenol concentrations obtained in fruit peel, regardless of extraction solvents, were between 92 % and 97 % (for TPC) and 72 % and 88 % (for TFC) of the total concentrations obtained from all the co-products (Tables 1 and 2). The order of TPC and TFC being peel > marc > juice.

**Table 1:** Effects of extraction solvents and in vitro digestion on total phenolic concentrations (mg GAE/100 mL) of extracts of pomegranate co-products

		TPC (mg GAE/100 mL)					
		In vitro digestion phase			*Significance level*		
Sample	Extract	Undigested	Gastric	Duodenal	Extract (A)	Digestion phase (B)	A*B
Juice	Water	45.59 ± 7.48^de^	121.27 ± 18.37^a^	88.25 ± 10.30^bc^	0.0198	<0.0001	0.7586
	50 % Ethanol	56.29 ± 2.69^de^	125.30 ± 10.73^a^	104.70 ± 4.20^ab^			
	Ethanol	40.33 ± 5.24^e^	109.70 ± 0.38^ab^	69.33 ± 3.95^cd^			
Marc	Water	136.20 ± 6.99^e^	472.51 ± 11.88^b^	381.53 ± 3.37^cd^	<0.0001	<0.0001	<0.0001
	50 % Ethanol	179.58 ± 19.73^e^	429.40 ± 13.53^bc^	335.60 ± 58.24^d^			
	Ethanol	14.65 ± 0.72^f^	761.83 ± 1.81^a^	497.20 ± 24.89^b^			
Peel	Water	2658.00 ± 14.28^c^	2693.58 ± 52.64^c^	2264.42 ± 15.59^e^	<0.0001	<0.0001	0.0685
	50 % Ethanol	2992.93 ± 26.83^b^	3185.00 ± 103.80^a^	2935.46 ± 32.57^b^			
	Ethanol	2458.03 ± 65.19^d^	2605.41 ± 44.03^cd^	2242.77 ± 50.38^e^			

Average values (±S.E) are presented. Rows and columns with different letter(s), per fruit fraction, are statistically significant different (*p* < 0.05)

*GAE* gallic acid equivalent

**Table 2:** Effects of extraction solvents and in vitro digestion total flavonoid concentrations (TFC, mg CE/100 mL) of extracts of pomegranate co-products

		TFC (mg CE/100 mL)					
		In vitro digestion phase				*Significance level*	
Sample	Extract	Undigested	Gastric	Duodenal	Extract (A)	Digestion phase (B)	A*B
Juice	Water	37.87 ± 5.04^b^	43.07 ± 1.17^ab^	8.79 ± 0.94^c^	0.6801	<0.0001	0.6317
	50 % Ethanol	36.07 ± 2.64^b^	43.39 ± 3.03^ab^	9.02 ± 1.31^c^			
	Ethanol	37.13 ± 2.22^b^	50.71 ± 7.57^a^	7.64 ± 2.39^c^			
Marc	Water	199.40 ± 40.95^ab^	182.50 ± 10.63^ab^	41.05 ± 6.89^c^	0.0255	<0.0001	0.5887
	50 % Ethanol	157.80 ± 11.95^b^	183.30 ± 13.96^b^	37.55 ± 4.96^c^			
	Ethanol	282.20 ± 57.92^a^	249.10 ± 54.73^ab^	62.06 ± 13.91^c^			
Peel	Water	1197.00 ± 29.77^c^	1385.00 ± 64^b^	309.64 ± 73.62^e^	<0.0001	<0.0001	0.0016
	50 % Ethanol	1505.00 ± 98.14^ab^	1602.00 ± 105.10^a^	340.08 ± 86.31^e^			
	Ethanol	844.80 ± 9.35^d^	953.90 ± 49.27^d^	193.59 ± 39.15^e^			

Average values (±S.E) are presented. Rows and columns with different letter(s), per fruit fraction, are statistically significant different (*p* < 0.05)

*CE* catechin equivalent

TPC and TFC measured in undigested extracts were generally significantly (*p* < 0.05) lower than the corresponding gastric digests of all the investigated co-products with the exception of water and 100 % ethanol extracts of marc, and the increments followed similar trends as observed for undigested samples in terms of solvent extracts and co-products, with TPC and TFC again in the order of peel > marc > juice. In contrast, TPC and TFC obtained from the duodenal digests were significantly (*p* < 0.05) lower than those obtained from the corresponding gastric digests regardless of extraction solvents for all fruit fractions (Tables 1 and 2). Despite the degradation of total phenolics in the duodenal digests, when compared with total concentrations obtained from undigested extracts (undigested vs. duodenal digest), TPC remained significantly higher in juice and marc extracts. However, peel undigested extracts had significantly (*p* < 0.05) higher TPC, with between 1.02–1.17-fold TPC than the corresponding duodenal digests (Table 1). This was also the case for total flavonoids with between 3.8 to 5.2-fold concentrations in undigested extracts compared to the corresponding duodenal digests (Table 2). Albeit, the trend of peel > marc > juice was maintained for both TPC and TFC (Tables 1 and 2) in the duodenal digests. As determined by two-way analysis of variance, significance levels (*p* < 0.05) clearly showed that changes in TPC in pomegranate juice and peel, and TFC in juice and marc were influenced by the combined effects of the choice of extraction solvent and the phase of in vitro digestion in this study.

There has been a rapid increase in presentation of data on the phenolic concentration and antioxidant capacity of pomegranate fruit [23, 24]. However, studies detailing the stability of the total antioxidant capacity after in vitro digestion in pomegranate are sparse. By measuring total phenolic concentration and antioxidant capacity of pomegranate fruit co-products after in vitro digestion it is possible to provide physiological relevant data in a quick and cost-effective manner [25]. From the practical point of view, the present study was based on the choice of extraction solvents which are suitable for human consumption as the phenolic-rich extracts could be explored further for possible formulations of health-promoting supplements for the food and nutraceutical industries or possible inclusion of pomegranate waste (peel and marc) extracts into pomegranate juice. The study showed that 50 % ethanol extracted more phenolics than water and 100 % ethanol extracts. According to Li et al. [11], phenolics in pomegranate fruit are often extracted in higher amounts in a combination of polar solvents.

The antioxidant activities were found to vary in the same manner as the phenolic concentration before in vitro digestion. This was expected because the fruit co-products, irrespective of the extraction solvents, did not share the same phenolic concentration. In addition, this is in line with the general consensus that the antioxidant property of many fruits, including pomegranates, is directly related to the presence of specific phenolic compounds [17, 24]. The observed antioxidant property of the investigated pomegranate extracts is probably attributed to the phenolic acids, flavonoids, punicalin and hydrolyzable tannins, including punicalagins, anthocyanins and ellagic acid derivatives [4, 7, 17].

Crucially, in this study, results have shown that bioaccessible (released) total phenolic concentration of pomegranate fruit peel, marc and juice are unstable throughout the in vitro digestive process. The instability of total phenolics in a simulated digestion has been documented [14, 25–27]. Specifically, extracts from the fruit co-products displayed largely increased total phenolic and flavonoid concentration after the gastric phase of in vitro digestion but declined after duodenal phase, albeit above the pre-digestion levels for most of the extracts. This is in agreement with previous research on other fruits which have consistently shown a similar pattern (as observed in this study) in total phenolics after in vitro digestion [25–27]. In comparison with the previous result on pomegranate juice after the gastric phase of in vitro digestion, Perez-Vicente et al. [15] reported no difference in total phenolic concentration in comparison to the native sample (undigested sample). This discrepancy might be related to difference the food matrix characteristics and in vitro conditions of digestion [28]. The authors, however, observed a general increase in individual anthocyanin concentration as a result of acidic pH of the gastric phase of digestion. In agreement with this current study, however, Sengul et al. [29] reported higher recovery of total phenolic compounds was observed after gastric digestion of pomegranate extract. This phenomenon has been attributed to increasing of the flavylium cation in the acidic solution of gastric phase of digestion [15]. Thus it could be assumed that increases observed in total phenolic concentration (of the fruit part extracts) at the gastric phase of in vitro digestion could be attributed to acidic hydrolysis of phenolics glycosides to their aglycons during gastric digestion [27].

It could be suggested that the observed decline in total flavonoid concentration impact on the decrease in total phenolic concentration at the duodenal phase of in vitro digestion. This could be attributed to degradation in the weak alkaline environment (pH 7.4) as phenolic compounds, in particular flavonoids, are highly sensitive to alkaline conditions [27]. This is in agreement with a previous study on pomegranate juice [15]. According to the authors, a decrease in phenolic compounds, in particular, anthocyanins (belonging to flavonoid group), at this phase of in vitro digestion was attributed to the transformation of the flavylium cation to the colourless chalcone at the alkaline pH of the medium. Accordingly, decreasing trend in total flavonoid concentration in the duodenal digests was in agreement with Mosele et al. [28], who also reported significant losses of flavonoids after the duodenal phase of in vitro intestinal digestion of pomegranate products. In comparison with other fruit, Kamiloglu et al. [30] reported recovery of 7–69 % of initial total phenolic concentration of dried fruits and mixtures after duodenal phase of digestion while Perez-Vicente et al. [15], Fazzari et al. [31] and Bouayed et al. [27] reported 20 % (for pomegranate), 27–29 % (for sweet cherries) and 44.6–62.7 % (for apples), respectively.

### Changes in antioxidant capacity after gastric and duodenal phases of in vitro digestion

**Fig. 3:**
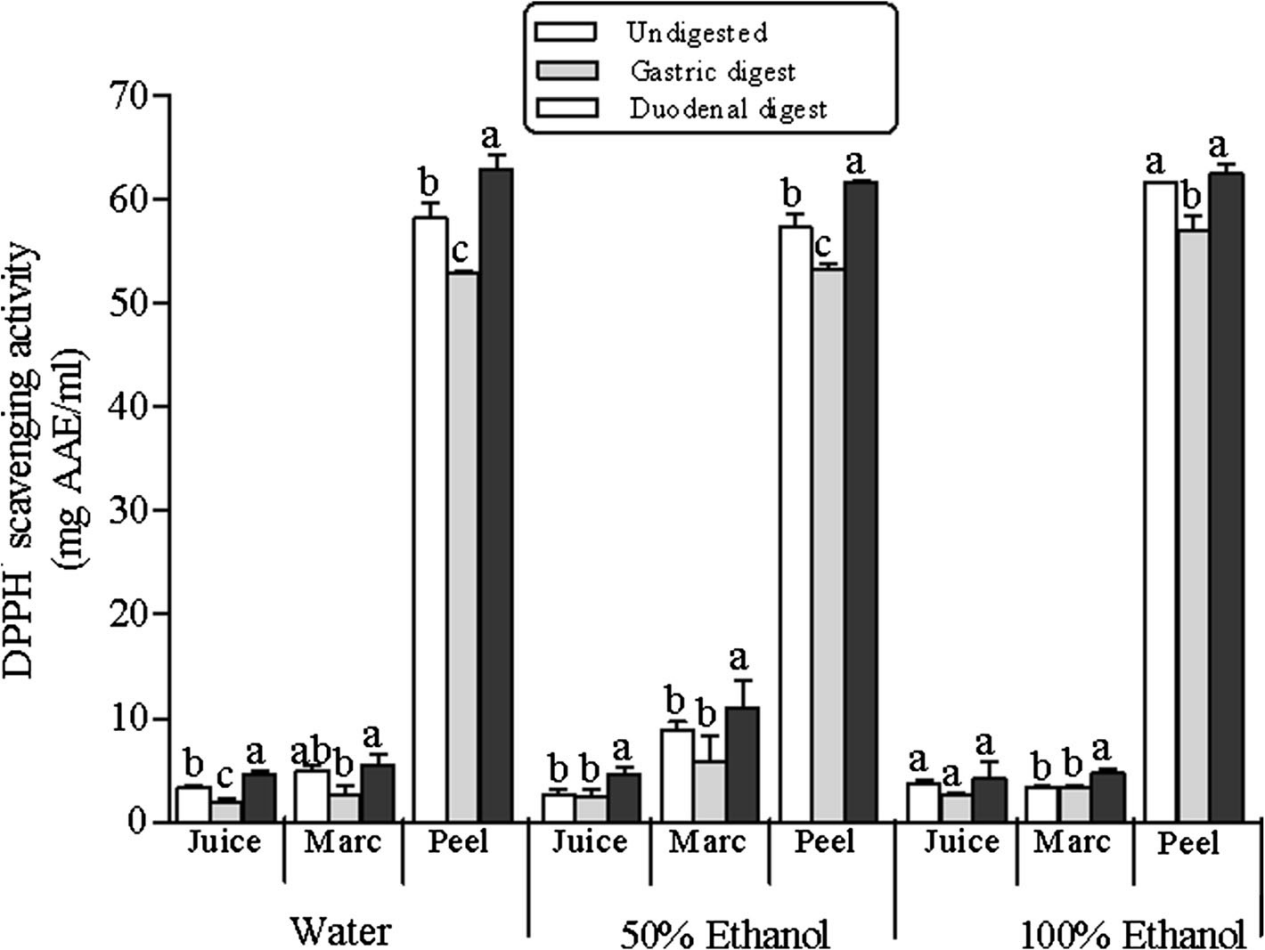
Changes in radical scavenging activity (RSA) during in vitro digestion model of water, 50 % ethanol and 100 % ethanol extracts of pomegranate peel, marc and juice. Average values (±S.E) of triplicate measurements are presented. Bars with different letter(s), per co-product for each solvent extract, are statistically significant different (*p* < 0.05). AAE, ascorbic acid equivalent

Prior to in vitro digestion, radical scavenging activity (RSA) reflected the levels of total phenolic concentrations in the investigated co-products, as evident by the order of peel > marc > juice (Fig. 3). Overall, the result showed that irrespective of the extraction solvent all the extracts were effective in scavenging the DPPH free radical (Fig. 3). The RSA decreased by 7–10 % in the gastric digests, with significant (*p* > 0.05) decreases observed for water extracts (juice, marc and peel), 50 % EtOH (peel) and EtOH (peel) (Fig. 3). However, in duodenal phase the ability of the extracts to scavenge the DPPH˙ radical increased significantly (*p* > 0.05) compared to those observed in the gastric digests, ranging between 5–18 % (Fig. 3). Interestingly, RSA was higher in extracts of duodenal digest in comparison to the radical scavenging activity exhibited by undigested extracts.

**Fig. 4:**
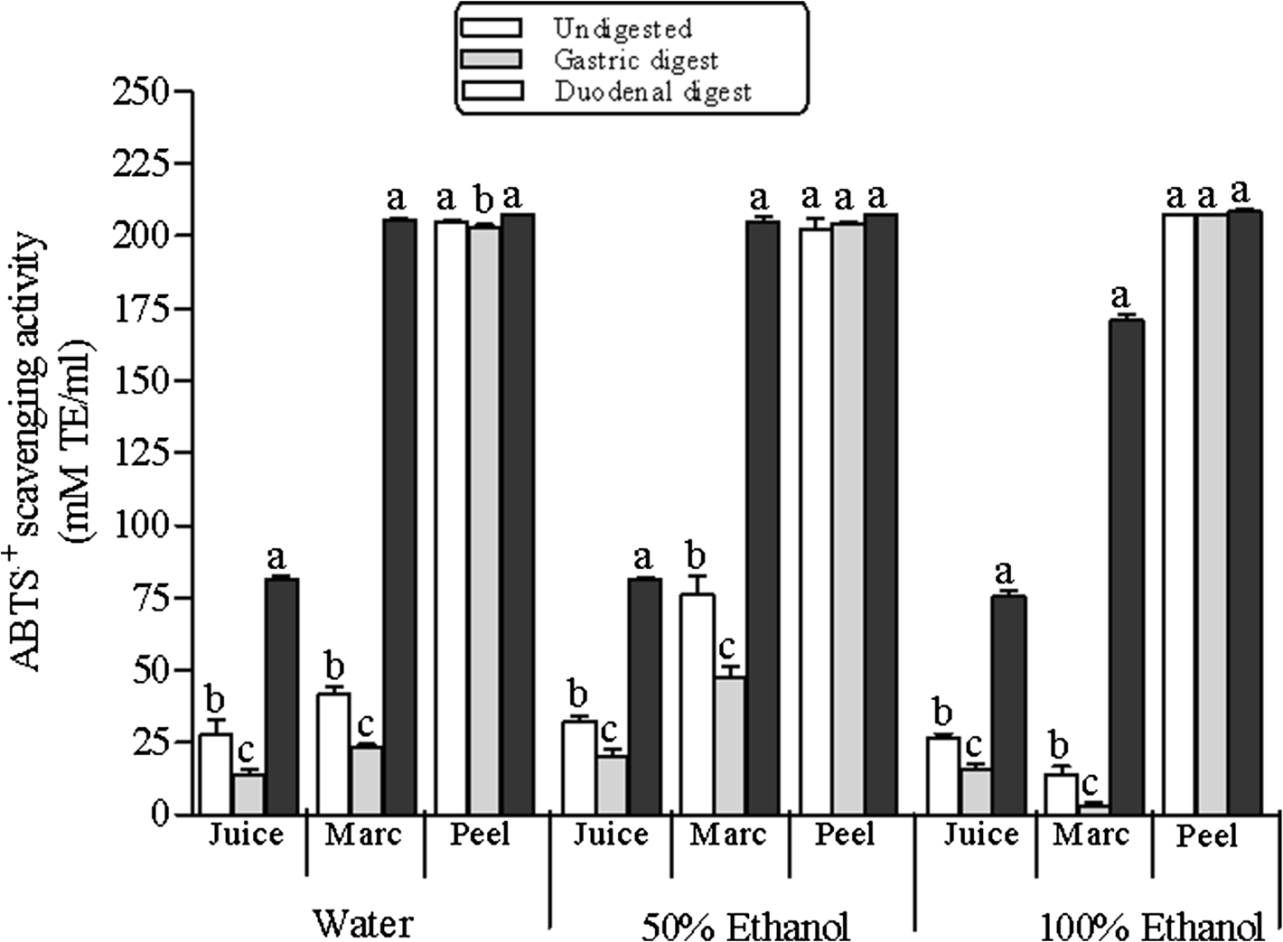
Changes in radical cation scavenging activity (RCSA) during in vitro digestion model of water, 50 % ethanol and 100 % ethanol extracts of pomegranate peel, marc and juice. Average values (±S.E) of triplicate measurements are presented. Bars with different letter(s), per co-product for each solvent extract, are statistically significant different (*p* < 0.05). TE, trolox equivalent

Generally, radical cation scavenging activity (RCSA) reflected the trend in TPC, again with the order being peel > marc > juice for all undigested extracts (Fig. 4). Again, the highest RCSA was exhibited by 50 % EtOH of undigested extracts and in vitro digests (Fig. 4). Amongst the co-products, peel extracts exhibited between 6–10-fold and 2.5–20-fold RCSA than juice and marc extracts, respectively. Similar to radical scavenging activity in DPPH assay, RCSA decreased significantly (*p* > 0.05) in the investigated extracts of gastric digests with the exception of peel 50 % EtOH and EtOH extracts, which showed no significant decline. However, RCSA exhibited by extracts of duodenal digests was higher than those exhibited by undigested and gastric digests. The main highlight was observed in all marc extracts in which between 5 and 75-fold radical cation scavenging activity was observed (Fig. 4).

**Fig. 5:**
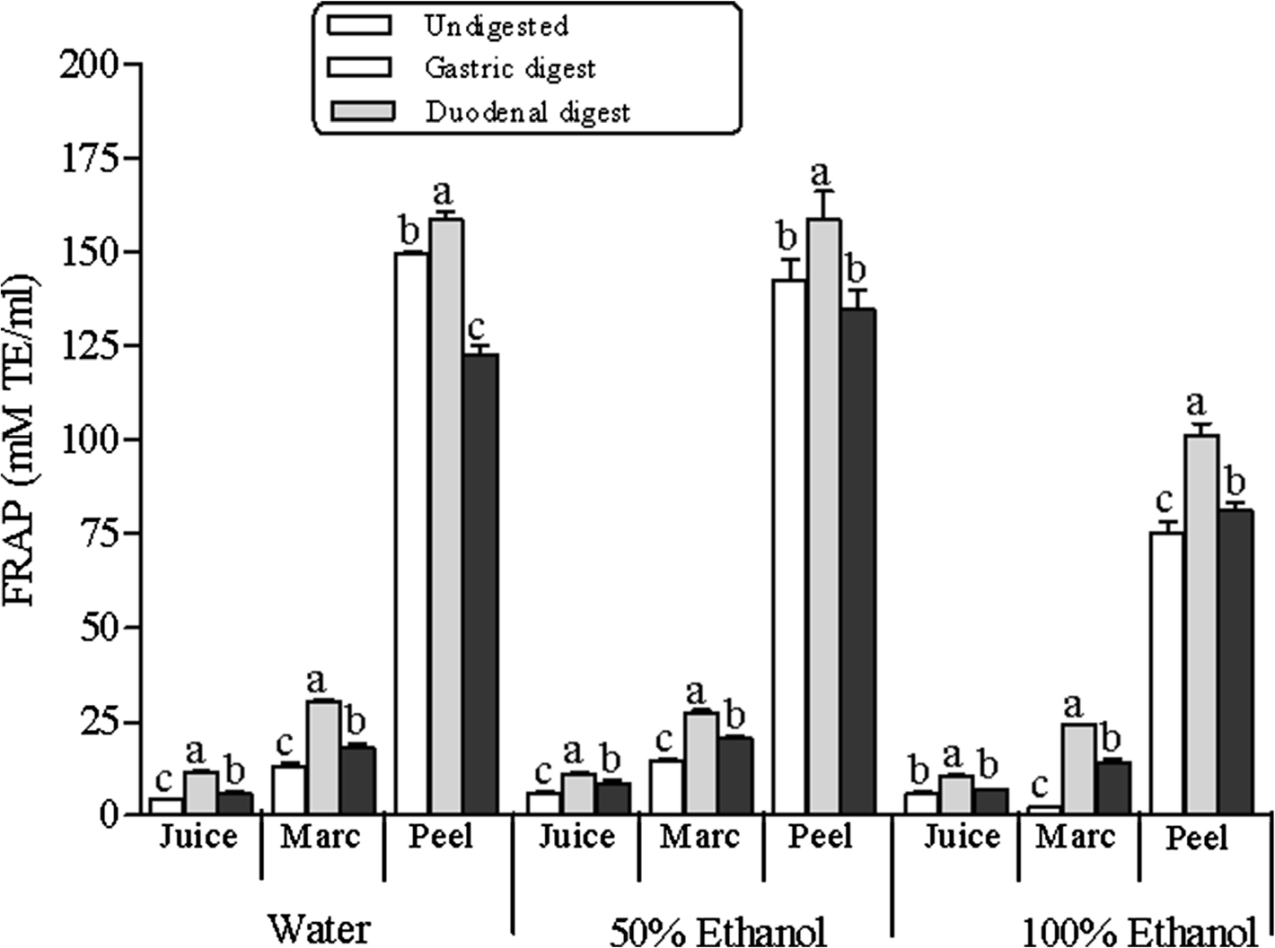
Changes in ferric reducing antioxidant power (FRAP) during in vitro digestion model of water, 50 % ethanol and 100 % ethanol extracts of pomegranate peel, marc and juice. Average values (±S.E) of triplicate measurements are presented. Bars with different letter(s), per co-product for each solvent extract, are statistically significant different (*p* < 0.05). TE, trolox equivalent

As observed in the anti-radical activities (DPPH˙ and ABTS^+^), the reducing antioxidant powers of the investigated extracts of pomegranate co-products were consistent with the total phenolic concentrations measured in undigested extracts (Fig. 5). Overall, 50 % ethanol and water extracts showed higher reducing power than ethanol extracts (Fig. 5). Furthermore, peel extracts showed between 5 to 30-fold reducing power than juice and marc extracts, with the activity again in the order of peel > marc > juice. Interestingly, contrary to the anti-radical activities measured by DPPH and ABTS^+^ assays, the FRAP values increased significantly in the gastric phase of digestion for all the extracts, perhaps as a result of the observed increase in phenolic concentration at this phase. However, the reducing powers decreased significantly (*p* > 0.05) by 10 to 26 % in the duodenal digests. Albeit, FRAP values remained relatively higher in duodenal phase compared to undigested extracts (Fig. 5).

This study has demonstrated that bioaccessible phenolics were able to reduce or scavenge free radicals (in the DPPH and ABTS^+^ assays) during a simulated digestion. The radical scavenging activities in both gastric and duodenal phases of in vitro digestion were even higher than those exhibited before being subjected to a simulated digestion, presumably due to the dependency of phenolic activity on pH of the digestion medium. For instance, high pH values (alkaline pH) have been reported to significantly increase phenolics scavenging ability [26]. It is believed that transition from acidic to alkaline environment enhances the antioxidant activity of phenolics by causing deprotonation of hydroxyl moieties present on their aromatic rings [27]. This buttresses the report by Lee et al. [32] that aglycones phenolics display an antioxidant power higher than their glycoside forms. Specifically, since radical scavenging activity is mainly dependent on the number and position of hydrogen-donating hydroxyl groups on the aromatic rings of the phenolic compounds [26, 33], it would thus be appropriate to evaluate antioxidant activity in the duodenal phase of in vitro digestion conducted in weak alkaline condition rather than in the gastric acidic environment.

The observed dynamics of the reducing power of the investigated extracts again could primarily be due to pH of the medium. The pH of a substance is known to affect racemization of molecules, possibly creating two chiral enantiomers with different reactivity [25]. As a result, some antioxidants could be rendered more reactive at acidic pH in the gastric phase and less reactive at alkaline pH during the duodenal phase of in vitro digestion [25], a trend observed in this current study. In addition, polyphenols are highly sensitive to alkaline conditions and do transform into different structural forms with different chemical properties [13, 34]. Since this was observed consistently across the investigated pomegranate extracts, it would be reasonable to assume that the overall loss in reducing power (between gastric and duodenal phases) in this current study could be as a result of the above-mentioned reasons during the duodenal phase of in vitro digestion at pH 7.4 (weak alkaline). In addition, with respect to their stability, one could assume that the phenolic antioxidants in the investigated extracts responsible for the ferric reduction in FRAP assay are fewer at the duodenal phase (compared to the gastric phase), transformed or impaired [25]. Furthermore, according to Wootton-Beard et al. [25], it could also be suggested that metabolites formed as a result of structural changes in the alkaline condition could have reacted differently in the FRAP assay.

Pearson correlation was used to investigate the relationships between polyphenolic concentrations and antioxidant capacity in the three assays investigated before digestion and at different phases of gastro-intestinal digestion (Table 3). In undigested samples, strong and positive relationships were revealed between TPC and TFC and the antioxidant activities measured in DPPH, ABTS^+^ and FRAP assays, with correlation coefficients (*r*^2^) ranging between 0.930–0.990 (Table 3). The intra-and interrelationships were equally strong and positive in the gastric phase of in vitro digestion between all the investigated parameters. As regards duodenal phase, again a strong and positive correlations were achieved among TPC or TFC and the radical scavenging activity (in DPPH) and antioxidant power (FRAP), while those between polyphenols (TPC and TFC) and radical cation scavenging activity in ABTS^+^ were moderately positive (Table 3). This suggests that while extracts of pomegranate co-products could be considered as a polyphenolic-rich source, the relationship between pomegranate phenolics and radical cation scavenging activity during the duodenal phase of in vitro digestion may not be associated with the pre-digested phenolic concentration.

**Table 3:** Pearson correlations between the studied total polyphenols and antioxidant capacity for extracts at different in vitro digestion phases

Sample	Variable	DPPH	ABTS	FRAP
*Undigested*	TP	**0.989**	**0.981**	**0.961**
	TF	**0.932**	**0.924**	**0.972**
*Gastric digest*	TP	**0.977**	**0.968**	**0.973**
	TF	**0.947**	**0.947**	**0.995**
*Duodenal digest*	TP	**0.976**	0.652	**0.978**
	TF	**0.947**	0.635	**0.996**

Values in bold are different from 0 with a significance level *P* = 0.05

## Conclusions

There is a large variation in the phenolic concentration and antioxidant capacity of the investigated fruit co-products. These results suggest that pomegranate waste (peel and marc) could be considered as a source of great interest to obtain pomegranate phenolic extracts for nutraceutical and development of value-added products. Findings from this study also showed that the concentration of pomegranate polyphenols and the antioxidant capacity during in vitro gastro-intestinal digestion may not reflect the pre-digested phenolic concentration. Thus, this study highlights the need to provide biologically relevant information on antioxidants by providing data reflecting their stability and activity after in vitro digestion.

## Abbreviations

%: percentage
°C: Degree celsius
ABTS: 2,2-azino-bis (3-ethylbenzothiazoline-6-sulphonic acid
CE: Catechin equivalents
DPPH: 2,2-diphenyl-1-picrylhydrazyl
EtOH: Ethanol
FRAP: Ferric reducing antioxidant power
mM AAE/mL: milliMolar ascorbic acid equivalent per millilitre
mM TE/mL: milliMolar trolox equivalents per millilitre
pH: Potential of hydrogen
RCSA: Radical cation scavenging activity
RSA: Radical scavenging activity
S.E: Standard error
TFC: Total flavonoid concentration
TPC: Total phenolic concentration
TPTZ: 2,4,6-tripyridyl-s-triazine
